# Supplementary figures and images for: Metabolic gene regulation by Drosophila GATA transcription factor Grain
Source: PLoS Genet. 2021 Oct 11;17(10):e1009855. doi: 10.1371/journal.pgen.1009855 (PMC8530363; doi:10.1371/journal.pgen.1009855)

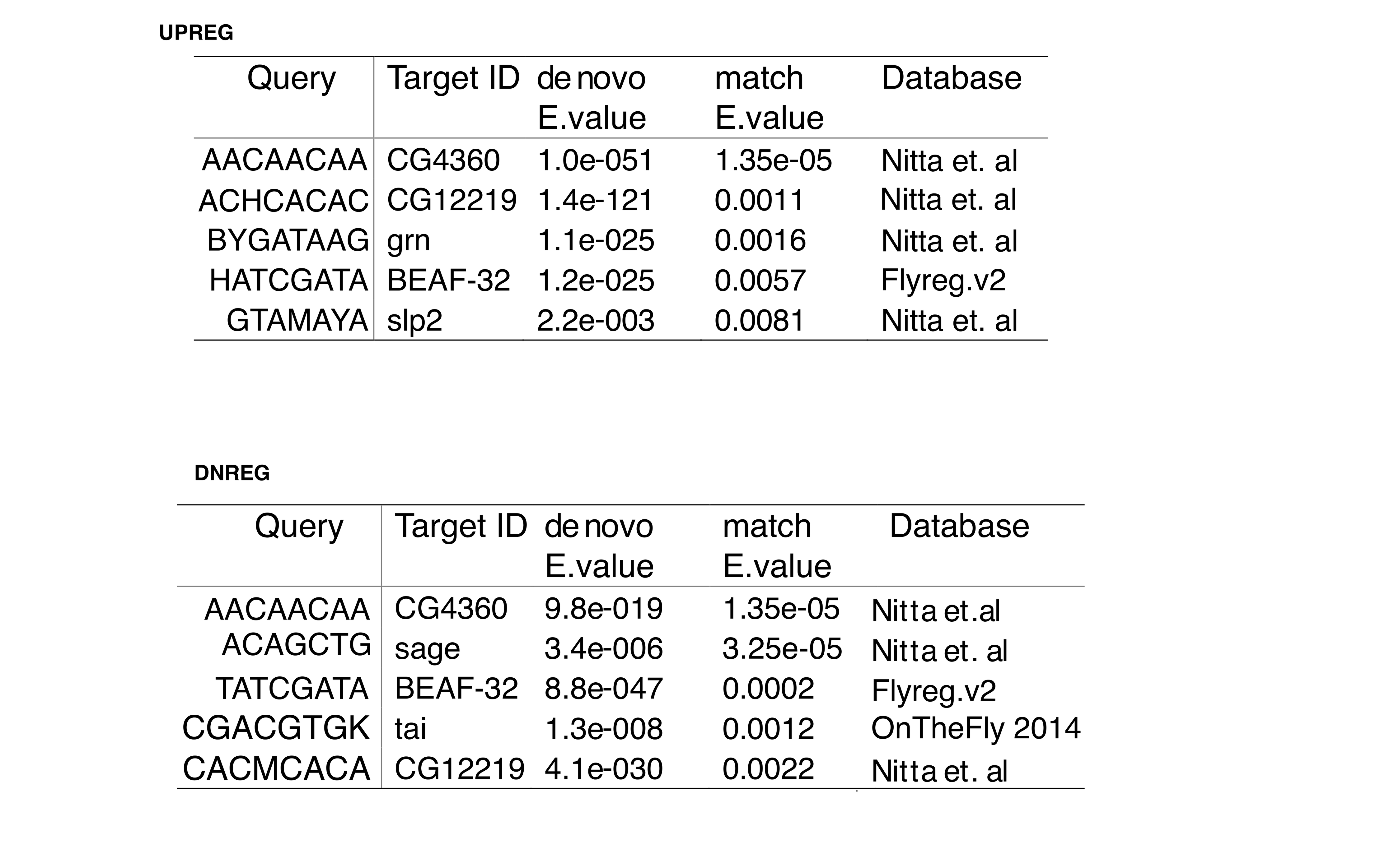

Supplement: S1 Table — (TIF) [file pgen.1009855.s001.tif]

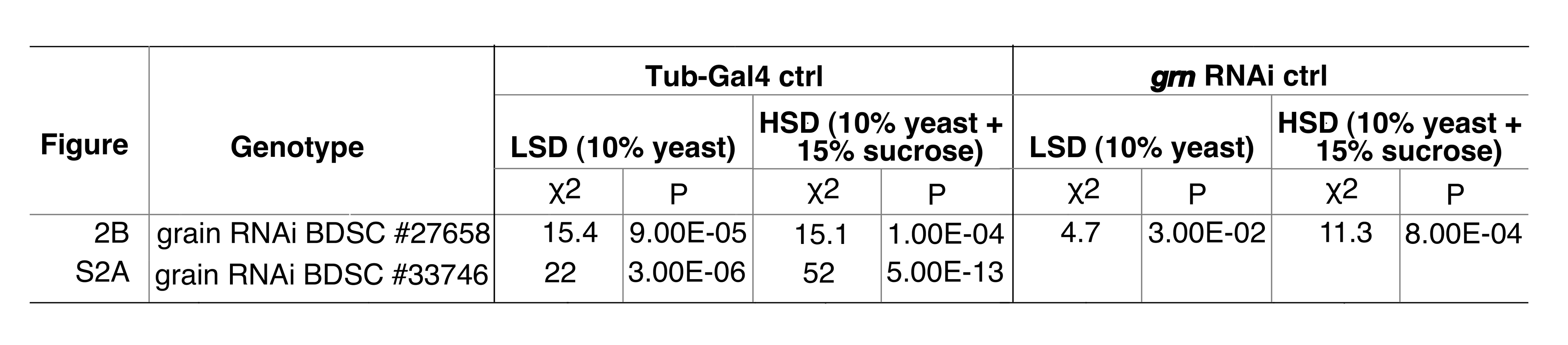

Supplement: S2 Table — (TIF) [file pgen.1009855.s002.tif]

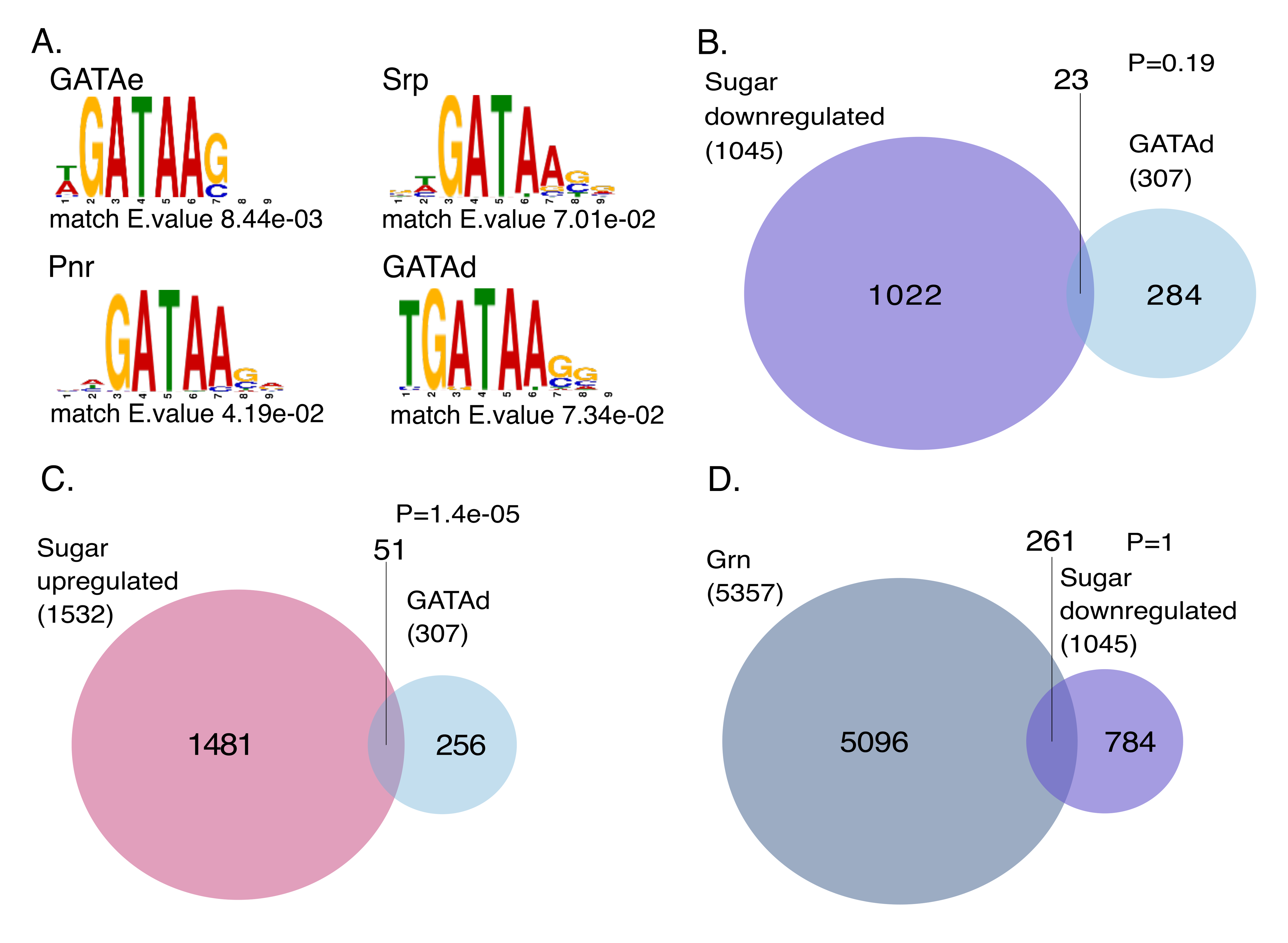

Supplement: S1 Fig — A. GATA TF (GATAe, Srp, Pnr, GATAd) database matches to de novo predicted binding motif in 1B and their statistics (TOMTOM). B. Venn diagram of GATAd direct targets (ChIP-seq, ENCODE dataset ENCSR245UCO) and genes upregulated on high sugar diet (RNA-seq, adj.p.val<0.05). C. Venn diagram of GATAd direct targets (ChIP-seq, ENCODE dataset ENCSR245UCO) and genes downregulated on high sugar diet (RNA-seq, adj.p.val<0.05). D. Venn diagram of Grain direct targets (ChIP-seq, ENCODE dataset ENCSR909QHH) and genes downregulated on high sugar diet (RNA-seq, adj.p.val<0.05). (TIF) [file pgen.1009855.s005.tif]

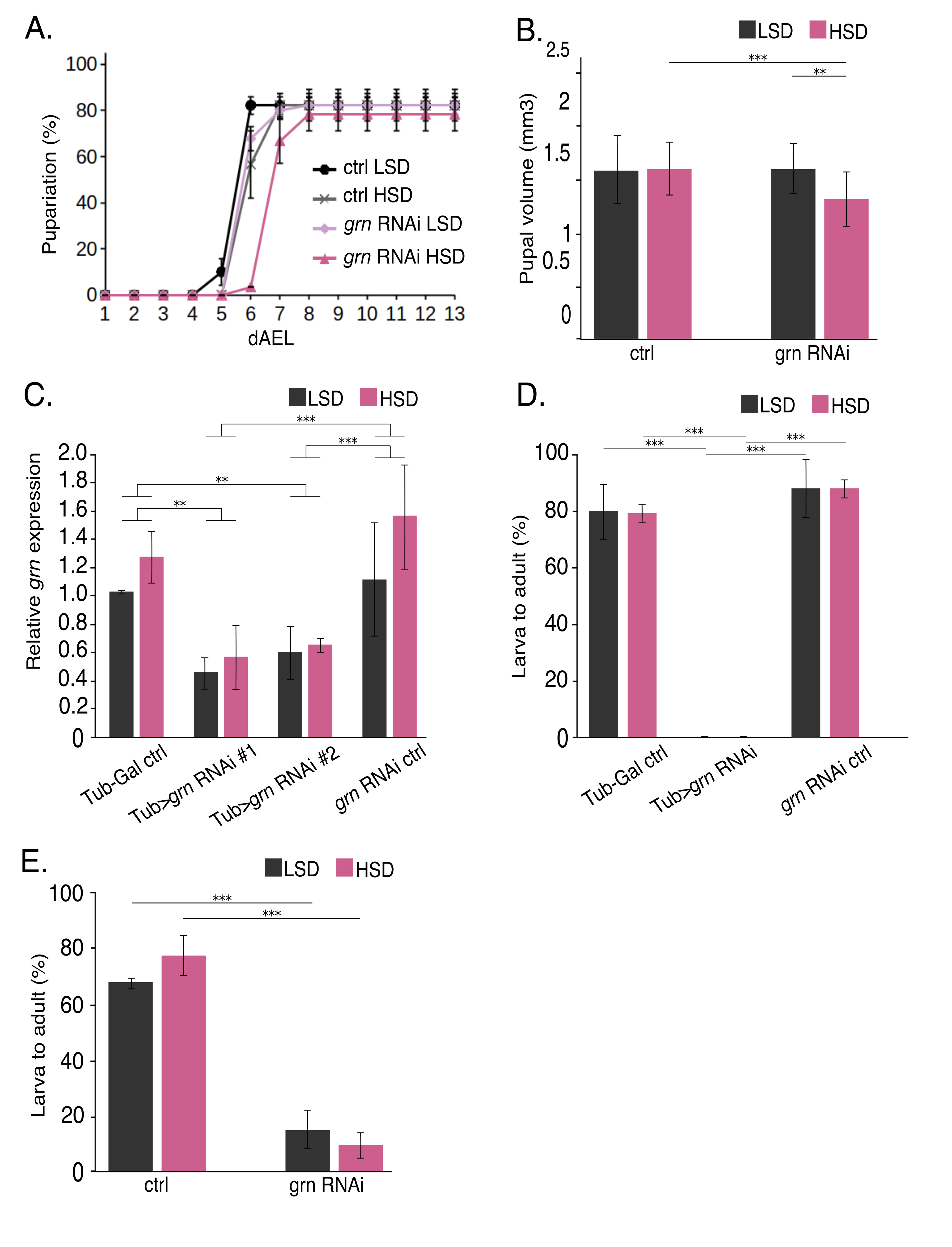

Supplement: S2 Fig — A. grain (grn) knockdown (Tub-GAL4) by an alternative RNAi-line (BDSC #33746) leads to developmental delay as compared to control (BDSC #31603) on both diets, but is more pronounced on a HSD. (N = 3 for control and grain RNAi on LSD, 2 for grain RNAi on HSD, 30 per replicate). B. grain knockdown (Tub-GAL4) by an alternative RNAi-line (BDSC #33746) leads to reduced pupal volume on a HSD as compared to control (BDSC #31603). (N = 30). C. Relative grain expression following Tub-GAL4-driven knockdown by two independent RNAi lines (#1: BDSC #27658; #2: BDSC #33746), compared to controls (Tub-GAL4>TriP control BDSC #31603 and UAS-grain RNAi BDSC #27658). D and E. Knockdown of grain (Tub-GAL4) by two independent RNAi (D: BDSC #27568, E: BDSC #33746) leads to increased pupal lethality on both experimental diets. (N = 4 in (D), N = 3 for control and grain RNAi on LSD, 2 for Grain RNAi on HSD in (E)). Data information: N indicates the number of biological replicates. Error bars display standard deviation. (A). Log-rank test (Df = 3), p.val.<0.01. (B): Two-way ANOVA found a significant interaction effect between diet and genotype (F (2,194) = 3.715, p.val = 0.02611). A Tukey HSD was performed with p-values indicated on the graph as indicated below. (C): Two-way ANOVA found no significant interaction effect between diet and genotype. Diet (F (1,16) = 5.040, p.val = 0.0393) and genotype (F (3,16) = 17.802, p.val = 2.37e-05) effects were found to be significantly different. A Tukey HSD was performed with p-values indicated on the graph as indicated below. (D): Two-way ANOVA found no significant interaction effect between diet and genotype. A genotype (F (2,16) = 368.184, p.val = 4.18e-14) effect was found to be significantly different. A Tukey HSD was performed with p-values indicated on the graph as indicated below. (E): Two-way ANOVA found no significant interaction effect between diet and genotype. Diet (F (1,7) = 7.067, p.val = 0.0325) and genotype (F (1,7) = 316.988, p.val = [file pgen.1009855.s006.tif]

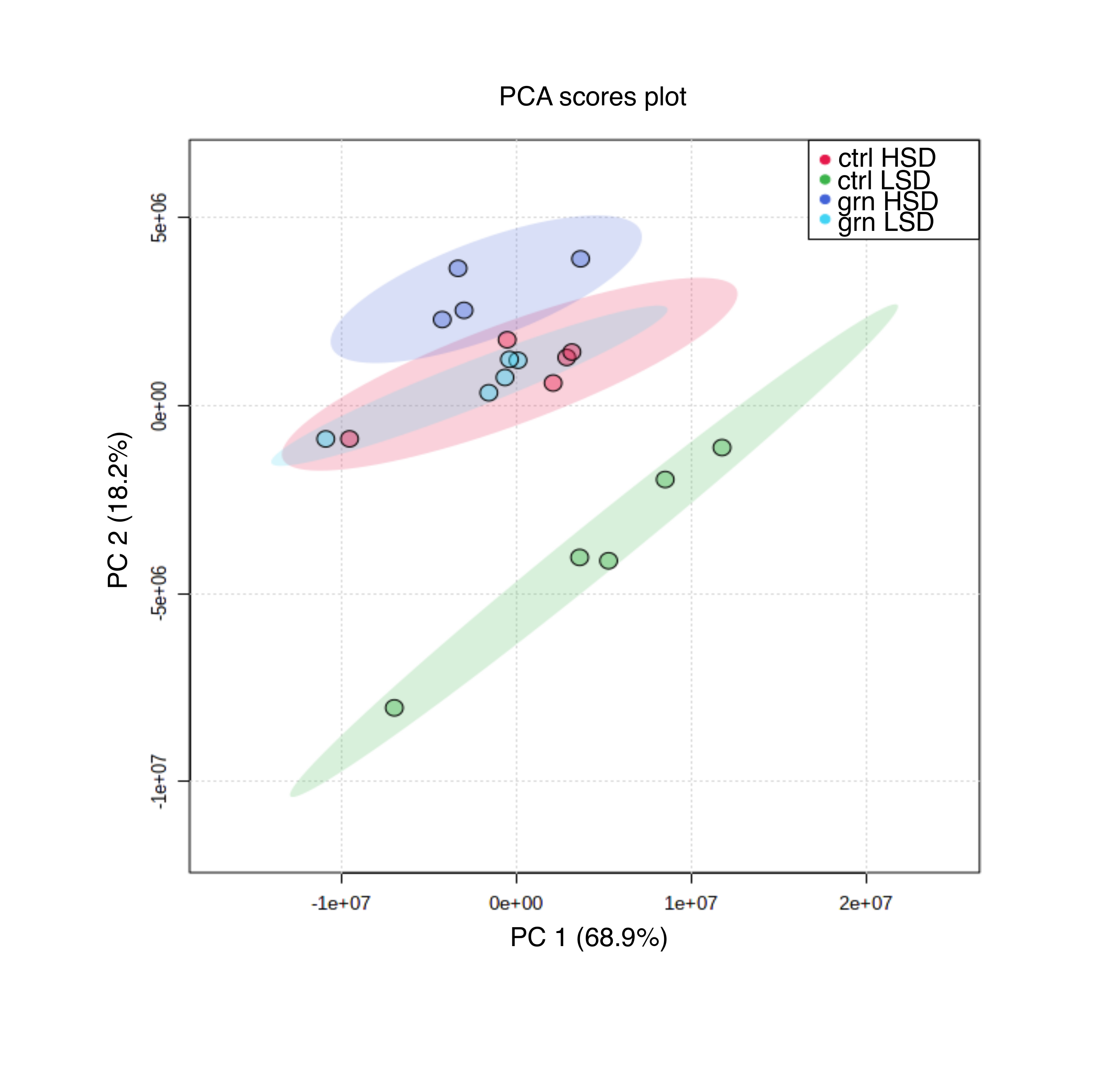

Supplement: S3 Fig — (TIF) [file pgen.1009855.s007.tif]

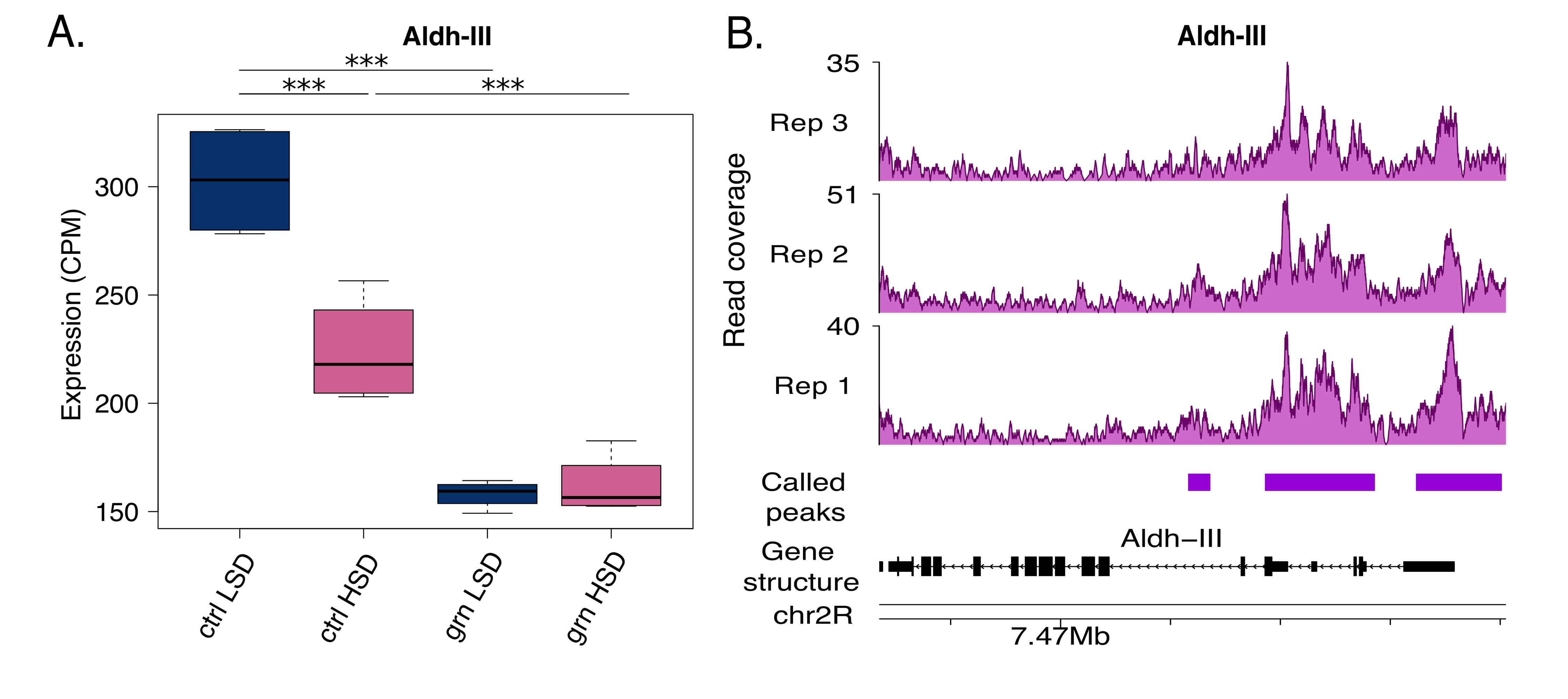

Supplement: S4 Fig — A. Expression of aldh-III (RNA-seq) is strongly downregulated by grain knockdown (Tub-GAL4>grain RNAi BDSC #27568) as compared to control (Tub-GAL4>TriP control BDSC #31603) 2nd instar larvae on both a low sugar diet and after 8 hours sugar exposure. (N = 4, 20 per replicate). ***adj.p.val<0.001. LSD: 10% yeast, HSD: 10% yeast + 15% sucrose. B. Binding profiles of Grain in aldh-III promoter (ChIP-seq, ENCODE dataset ENCSR909QHH) showing that aldh-III is a direct target of Grain. Purple bar indicates the called peak are in Grain ChIP-seq. (TIF) [file pgen.1009855.s008.tif]

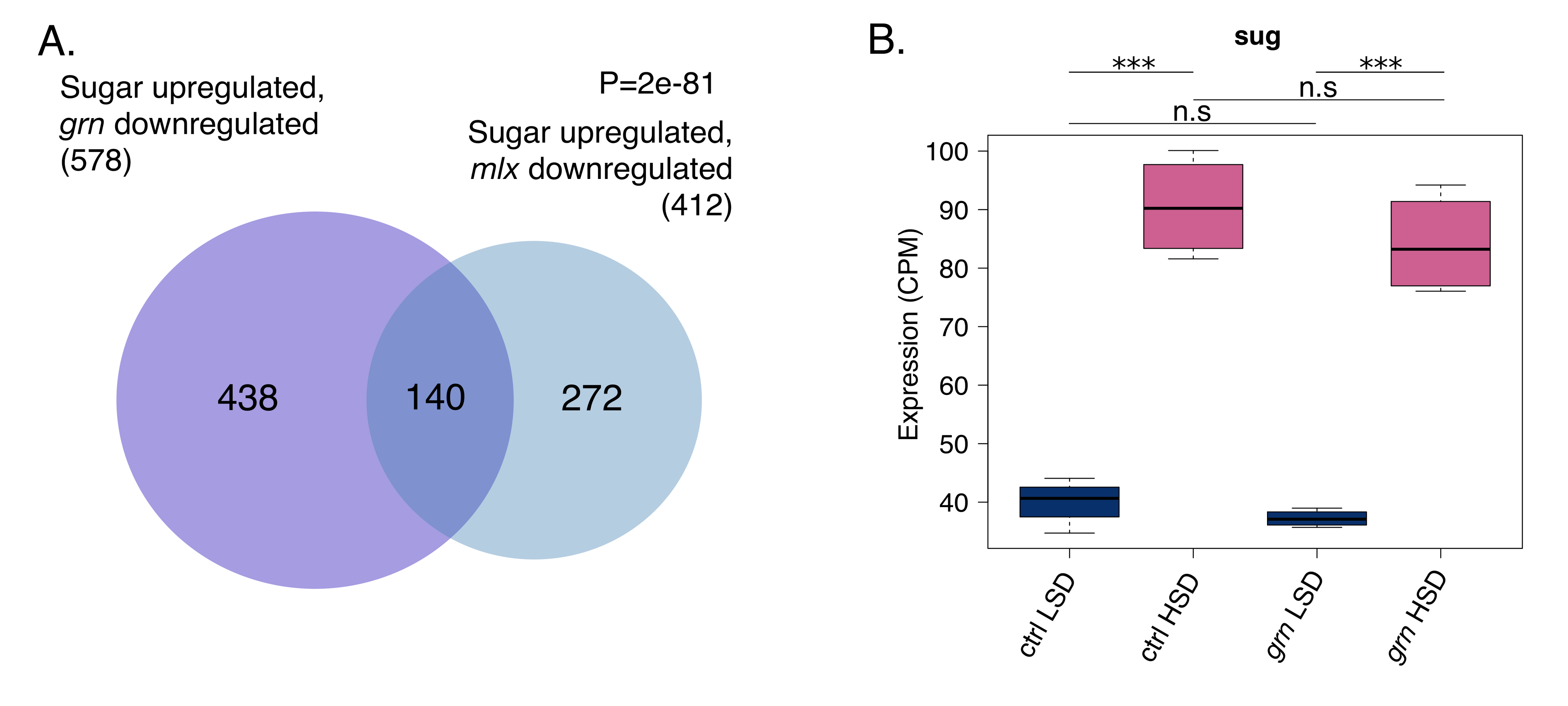

Supplement: S5 Fig — A. Venn diagram displaying the overlap between genes that are upregulated (RNA-seq) in 2nd instar larvae after 8 hours of high sugar diet in Grain- and Mlx-dependent manner (adj.p.val<0.05). B. Expression of sugarbabe (RNA-seq) is not affected by grain knockdown (Tub-GAL4>grain RNAi BDSC #27568) as compared to control (Tub-GAL4>TriP control BDSC #31603) 2nd instar larvae on both a low sugar diet and after 8 hours sugar exposure. (N = 4, 20 per replicate). CPM: counts per million. (TIF) [file pgen.1009855.s009.tif]

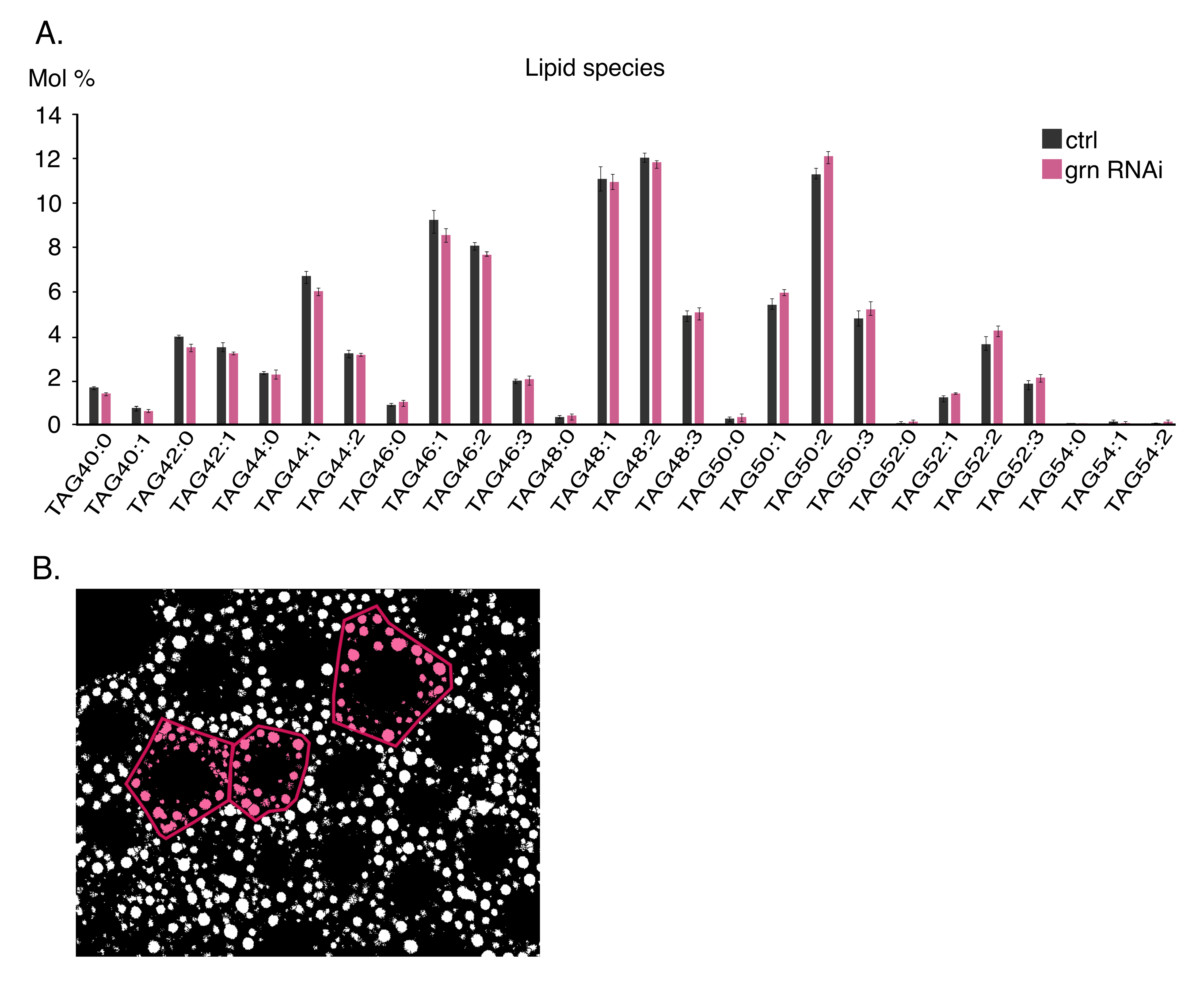

Supplement: S6 Fig — A. Composition of TAG lipid species (mol% profiles measured by mass spectrometry-based lipidomics) in Grain depleted (grn) (Tub-GAL4>grain RNAi BDSC #27658) and control (Tub-GAL4>TriP control BDSC #31603) pre-wandering 3rd instar larvae after being fed a low sugar diet with 1% glucose for 24 hours. (N = 4, 15 larvae per sample). B. Example of fat body lipid droplet identification by the Analyze Particles function in FIJI of Grain depleted (Tub-GAL4>grain RNAi BDSC #27658) and control (Tub-GAL4>TriP control BDSC #31603) fat bodies of early 3rd instar larvae raised on a diet either low or high in sugar. (TIF) [file pgen.1009855.s010.tif]

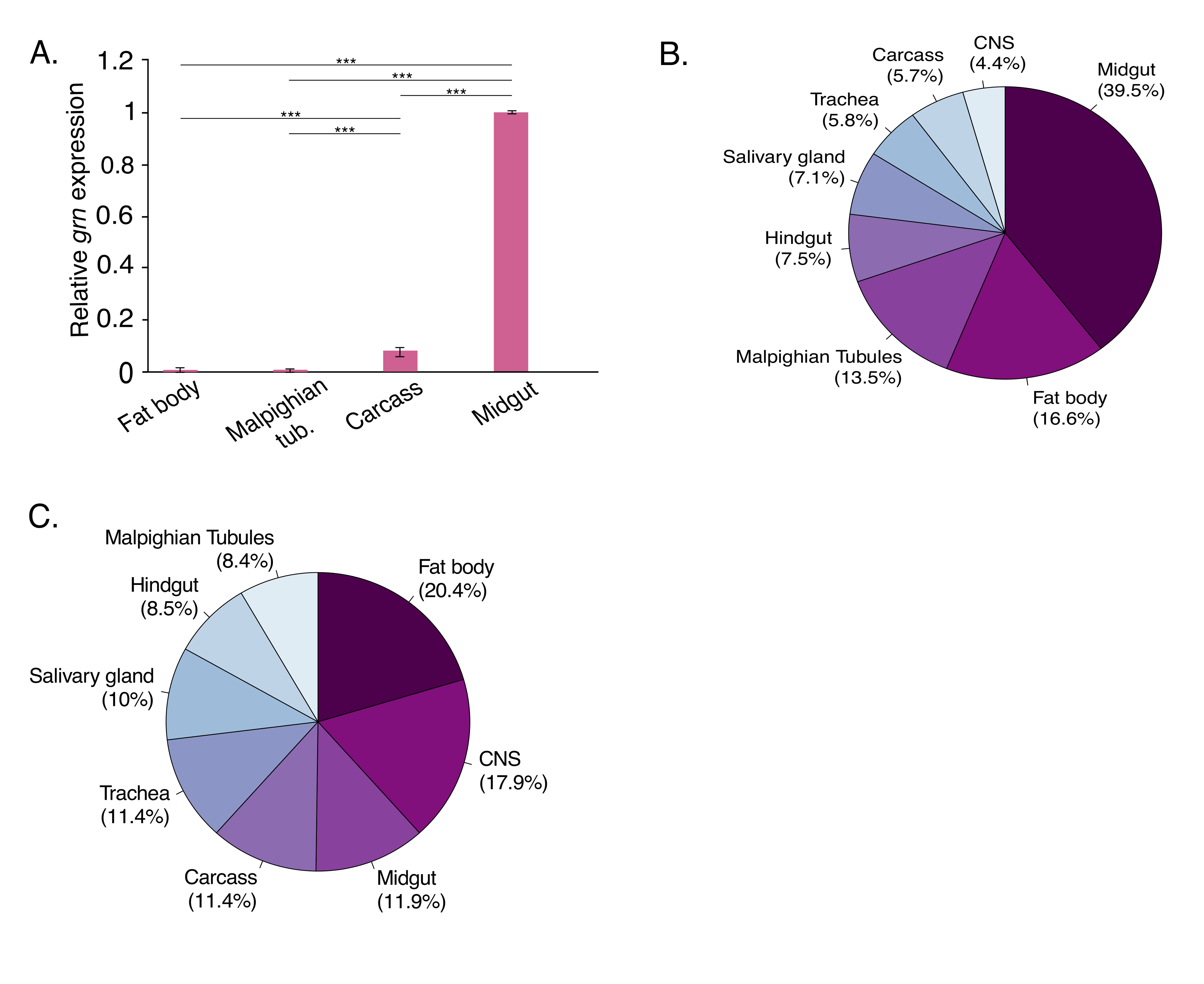

Supplement: S7 Fig — A. Relative grain mRNA expression in larval fat body, Malpighian tubules, carcass, and midgut. Midgut samples were used as reference samples, and expression of CDK7 was used for normalization. (N = 4, tissues from 5 larvae per sample). B. Tissue specific expression of differentially regulated, sugar-responsive Grain target genes (RNA-seq, adj.p.val <0.05, LFC +/-0.5). The relative expression of sugar-responsive Grain target genes across tissues was calculated based on FlyAtlas2 expression data. C. Tissue specific relative expression of all Drosophila genes present in FlyAtlas2. The relative expression of selected genes across tissues was calculated based on FlyAtlas2 data. Data information: N indicates the number of biological replicates. Error bars display standard deviation. (A). One-way ANOVA found a significant interaction effect between tissues (F (3,12) = 11628, p.val < 2e-16). A Tukey HSD was performed with p-values indicated on the graph as follows: *p.val<0.05, **p.val<0.01, ***p.val<0.001. (TIF) [file pgen.1009855.s011.tif]
