# Supplementary material for: Metabolic gene regulation by Drosophila GATA transcription factor Grain
Source: PLoS Genet. 2021 Oct 11;17(10):e1009855. doi: 10.1371/journal.pgen.1009855 (PMC8530363; doi:10.1371/journal.pgen.1009855)
Supplement: Attachment — Submitted filename: Kokki_Lamichane_point by point response.docx [file pgen.1009855.s012.docx]

**Kokki, Lamichane et al., point-by-point response**

**Reviewer #1: PGENETICS-D-21-00232**

Metabolic gene regulation by Drosophila GATA transcription factor Grain

Kokki et al.

In this study Kokki et al. describe characterizing genes whose regulation by the Grain transcription factor (TFs) are affected by sugar levels. They start by re-evaluating a previously published data set identifying sugar-regulated genes in 2nd instar Drosophila larvae. In silico motif identification led to their study of the role of Grain (grn) TF. They carried out ubiquitous knockdown of grain and identified developmental delays and reduced pupal volume. They then show target genes that are downregulated in grn-RNAi, under high (HSD) and low (LSD) sugar diets. Subsequent analyses identify a number of metabolic alterations in grn-RNAi animals. The authors next probe if other TFs might act similarly and identify 66 genes that are downregulated when either sugarbabe or grain are depleted. The effects with grain were most pronounced on LSD. The authors then go on to investigate if both Sug and Gr might regulate the same genes and show overlapping binding sites in promoters of a number of genes. Finally, the authors show that lipogenesis and lipid droplet area is affected in grn depleted animals.

Overall this was a well written and thorough analysis that reveals new information about transcriptional targets of Grain under difference diet conditions. I have some minor concerns and suggestions. It seems that the information will be valuable to scientists interested in how diet contributes to changes in gene expression. I urge them to do follow up studies on the developmental phenotypes, and whether they can identify roles for any of the target genes in those processes.

Comments and questions:

1. The authors highlight the effects seen in LSD and seem to downplay that some genes are affected under HSD as well. Given that they conclude in talking about Grain and Sug, perhaps they could also focus on some HSD targets, at leats in the discussion.

*Our tissue specific analysis has now provided more insight into the roles of Grain in both HSD and LSD conditions, and therefore the emphasis on HSD vs. LSD should be more balanced (see Figure 9 and 10).*

2. Fig. 3 shows that a number of glycolytic genes were downregulated in grn-RNAi conditions, either under LSD or both. They followed this up with LC-MS metabolomics. Given that some glycolytic genes were also downregulated on a HSD do the metabolomics data sets show similar effects? Why are some metabolites significantly different between HSD and LSD in grn-RNAi while others are not?

*The majority of metabolites showed most prominent changes in LSD conditions, being consistent with the overall trend seen in the RNAseq data. As the Reviewer correctly points out, there are some important exceptions from this trend, including Glucose levels, which are elevated in both diet conditions. However, our current data does not allow us to reliably conclude about the underlying reasons of levels of individual metabolites.*

3. Fig. 3B shows a prediction of the tissues in which they believe the genes are altered, but without experimentally determining this, it seems this is not a robust data set.

*We have now significantly expanded the experimental tissue-specific analysis of Grain target regulation (see Figure 9 and 10). Former Fig. 3B carries now less weight and is presented as a supplementary material (Figure S7).*

4. Fig. 6 shows a comparison of the transcriptional profiles of grn-RNAi and knockdown of sugarbabe, which is another sugar-responsive TF. In Fig. 6B, the data sets for the two control groups are not totally consistent. Do the authors have any thoughts on that? Also, it would be easier to compare the effects of sugar diet if, for example, the control and experimental LSD were side by side.

*Exact comparison is difficult based on the heatmaps as the Grain and Sug datasets have been normalized separately, which influences the color scales. While a majority of genes show similar sugar-responsive trends, the Reviewer is right that there are individual genes whose sugar responsiveness differ. In global gene expression analyses it is common to observe this kind of sporadic differences in different genetic backgrounds. We prefer to keep the sample order as it is, since this way it is consistent with the other figures in the manuscript.*

5. Fig. 6D and E are reversed. Why are the data for sug not included with grn for the genes that they propose are being co-regulated?

*Thank you for pointing out the mislabeling, which has now been corrected. As suggested, we have now added the lipogenic gene expression data as a reference (revised Figure 7).*

6. The authors claim in their abstract that Grn and Sug both control lipogenic genes, yet the data is only shown for grn. In their discussion they go so far as to suggest that Sug could be a cooperative binding partner for Grain. I don’t feel their data support that statement since they show their effects on target genes are different under LSD/HSD and no other relevant data Sug effects are shown. According to their paper these two TFs regulate target genes under very different conditions. I believe they try to explain this in the discussion, but I think they also need to soften their interpretation of this data set.

*The role of Sug in regulating lipogenic gene expression has been shown earlier (Mattila et al., 2015), but we have now included data on sug-dependent lipogenic gene regulation as a reference for the Grain data (Figure 7). In light of the new tissue specific data, we have modified our Discussion accordingly, presenting alternative models to explain the data and being careful not to make over-interpretations.*

**Reviewer #2**: In this interesting manuscript, Kokki and Lamichane et al. use a bioinformatic approach to identify GATA response elements in the promoters of genes that are co-regulated by dietary sugar in Drosophila. They find that the identified response elements closely match DNA sequences bound by the GATA family member grain. Whole-animal loss of grain leads to reduced survival of larvae to the pupal stage, a delay in pupation, and reduced pupal volume in animals on a high sugar diet. RNA-sequencing in animals with ubiquitous grain knockdown shows reduced expression of a number of glycolytic enzymes in grain knockdown larvae on a low sugar diet. Somewhat paradoxically, levels of glycolytic intermediates and TCA cycle components are elevated in larvae with ubiquitous grain knockdown, with strongest effects occurring on low sugar. The authors also show that grain participates in a network of transcription factors that are activated in response to dietary sugar. Mlx is a positive regulator of grain and grain positively regulates expression of cabut and smox on a low sugar diet. Furthermore, grain and the transcription factor sugarbabe bind to overlapping regions on promoters of genes such as ACC, FASN1 and ATPCL. This paper adds to our understanding of nutrient sensing and signaling, and it would benefit from additional work to determine what tissues grain acts in to regulate glucose homeostasis.

Major comments.

1. Because grain mutants exhibit embryonic lethality, the authors used two different RNAi transgenes to knockdown grain ubiquitously, demonstrating effects on survival on high sugar. What is the degree of grain knockdown with these transgenes?

*As requested, we show the expression of grain mRNA after knockdown with the two independent RNAi lines (Figure S2C).*

2. In Figure 3B, the expression pattern of genes regulated by sugar in a grain-dependent manner is assessed using a computational approach (FlyAtlas2 queries). Midgut and fat body are the two dominant tissues. What is grain expression in these tissues?

*We have now significantly expanded the tissue specific analysis of Grain expression and function. Our data shows that Grain displays highest expression in the midgut, specifically in the anterior-most area (Figures 9 and 10, Figure S7).*

3. Is grain expression regulated by dietary sugar in a tissue-specific manner?

*We have analyzed grain expression in four larval tissues and have observed sugar-activated expression in the fat body and Malpighian tubules and constitutive expression in the midgut and carcass (Figure 9).*

4. RNA-sequencing of whole larvae is used to examine gene expression changes in response to ubiquitous grain knockdown. Does grain knockdown lead to equivalent changes in putative target genes (Hex-A, Pgi, AcCoAs) in midgut and fat body?

*We have done the tissue-specific analysis of Grain target genes and show downregulation of target genes in both midgut and fat body (Figures 9 and 10) albeit with different sugar responsiveness.*

5. The transcription factors Smox and cabut are positively regulated by grain (Figure 5). Can forced expression of either of these rescue glycolytic or lipogenic gene expression in larvae with grain knockdown?

*We tried performing the genetic epistasis experiment, as suggested. For the experiment, we tried recombining UAS-Smox/Cabut; UAS Grain RNAi (they are all in the 3^rd^ chrom.), but unfortunately did not obtain viable recombinants. Therefore the we could not perform the experiment during the time frame of the revision.*

6. Does loss of grain alter expression of sugarbabe?

*sugarbabe expression is unchanged in Grain-depleted animals. This data is now shown as Figure S5B.*

7. Loss of grain leads to late larval/early pupal lethality on high sugar but not low sugar (Figure 2). However, changes in glycolysis and TCA cycle intermediates (Figures 3, 4 and 6) and gene expression occur primarily on low sugar. Might these apparent discrepancies in sugar dependency relate to distinct sets of genes that drive the transition to metamorphosis rather than metabolism? Can the relatively minor effect sizes on metabolite levels observed with loss of grain explain the high sugar diet lethality?

*Our tissue-specific data reveals two modes for Grain function: constitutive (midgut) and sugar responsive (e.g. fat body). Grain displays highest expression in the anterior midgut, where it regulates lipogenic gene expression (Figure 10). Grain expression is sugar activated in the fat body, where it contributes to sugar-responsive expression of known sugar tolerance genes, such as cabut (Figure 9). The data is consistent with a model that the metabolic phenotypes on LSD and survival defects on HSD reflect these tissue-specific activities, respectively. However, detailed characterization of the physiological contribution of individual target genes remain to be done in future studies.*

Minor comments.

1. Throughout the manuscript, grain is inconsistently capitalized. The FlyBase gene name is “grain”, so it should be all lowercase. The authors may want to perform a similar check on other gene names in the paper.

*Grain (capitalized) refers to protein, grain (ital.) refers to gene. We have now checked the consistency of the naming throughout the manuscript.*

2. In the legend for Figure 6, the text describing panels D and E is swapped.

*Has been corrected.*

3. Line 160-161, a verb (“was”) is missing.

*Has been corrected.*

**Reviewer #3**: This is an interesting manuscript that identifies Grainy as a novel regulator of transcription in response to dietary sugar in Drosophila. Over the last years, the Hietakangas lab has been piecing together the components of the transcriptional network that respond to dietary nutrients using the fly as a model organism, and this manuscript is an interesting and useful addition towards generating a complete picture of how an animal responds to diet. Since Grainy is evolutionarily conserved to humans, it also raises the possibility that the human genes may have similar roles in terms of transcription and metabolism. Hence I think this manuscript will be of interest to a broad audience of people studying metabolism and transcription. The data are also of good quality.

That said, there is one major issue that I think should be addressed prior to publication. I see a disconnect between the clear metabolic consequences of Grn loss-of-function and the very mild transcriptional effects that are being reported here. I find it convincing that Grn regulates metabolism. For instance, the increases in metabolites on a low-sugar diet upon Grn loss-of-function shown in Fig 4 are clear and significant, as is the drop in survival on a high-sugar diet shown in Fig 1B and the reduced TAG levels shown in Fig 7A.

However, almost all of the transcriptional effects being shown, especially in the HSD, are extremely small in magnitude, and often not statistically significant, so that it is unlikely this would carry over to a detectable change in the corresponding protein levels. (In the HSD upon Grn loss-of-function, Hex-A in Fig 3C only drops by 5% and it’s not significant, Gapdh1 doesn’t drop, Gapdh2 doesn’t drop, PyK doesn’t drop, cbt in Fig 5 also only drops by 5% and not significantly.) If protein levels aren’t changing, this can’t explain the metabolic phenotypes. I think this issue needs to be resolved. In sum, I think it would be important to show at least one target gene that is changing at the protein level that can link the transcriptional effects to the metabolic ones.

*Thank you for the feedback. We have now addressed the tissue-specific roles of Grain and believe the new data addresses the concerns raised (see below for details).*

I can think of several different reasons for this disconnect, but I can’t guess which one is correct, so I mention several of them:

1. Much of the manuscript focuses on the transcriptional changes happening in the HSD condition. I assume this is because historically the authors identified Grn by looking at the HSD gene expression data (Fig 1). However, almost all the transcriptional data, the metabolite data, and the metabolic data show almost no effect of Grn loss-of-function in the HSD. Instead, all the phenotypes are more pronounced in the LSD. The only outlier that I can see is the survival curve show in Figure 2B which shows a survival defect in the HSD and not the LSD. Given that this does not seem to reproduce with a 2nd independent RNAi (Suppl. Fig. 2A), it makes me wonder if maybe the survival effect in main Fig 2B is an off-target artefact that is a red-herring causing the authors to focus much of the storyline on HSD? I think if the phenotype shown in Fig 2B cannot be reproduced with an independent RNAi, it is advisable to remove it. The metabolic consequences of Grn loss-of-function don’t necessarily need to lead to death to be biologically meaningful.

*Based on the new tissue specific data (Figures 9 and 10), it appears that there are two separate modes of Grain function, sugar-responsive and constitutive, which dominate in different tissues. grain expression is highest in the midgut, but it is not activated by sugar. On the other hand, fat body and Malpighian tubules show sugar-responsive grain expression. Moreover, in the fat body grain is necessary for the sugar-responsive upregulation of known determinants of sugar tolerance, such as cabut.*

*Regarding the lack of survival defect in the independent line, we would like to point out that it is common in these sugar-dependent growth/survival phenotypes to see quantitative differences between RNAi lines. I.e. weaker RNAi lines only causing delay and impairment of growth, while the stronger one causing an additional survival defect.*

2. All the transcriptional data show a larger magnitude effect upon Grn loss-of-function in the LSD compared to the HSD. Why is that? One option is that Grn becomes more activated (e.g. post-translationally) in the LSD condition. At first glance, this doesn’t seem to fit with the fact that some genes which look like Grn targets are increasing in the HSD compared to the LSD (e.g. Hex-A, cbt). However, all these genes increase in expression also when Grn RNAi animals are shifted from LSD to HSD (ie the 3rd to the 4th box plot in Figures 3C, 5A, 5C, etc). This means that their induction upon HSD is Grn independent and due to other transcription factors. Instead, all these target genes drop more upon Grn loss-of-function in the LSD condition, indicating that Grn is more active in the LSD. Hence maybe the focus of the manuscript in terms of linking transcriptional changes to metabolic changes should be in the LSD condition.

*This is indeed the case that most Grain-dependent gene expression changes are more prominent on LSD. We agree with the interpretation that the sugar-induced expression in this setting is driven by other TFs. Our new tissue-specific analysis brings more clarity into this issue, by showing tissue specificity in Grain target gene regulation, including sugar responsive function in the fat body, but not in the midgut (Figures 9 and 10). In light of the new data, we have now significantly revised our conclusions and hopefully brough more clarity into the sugar-dependent and independent roles of Grain.*

For instance, the authors write

“Interestingly, cabut expression was significantly downregulated upon Grain depletion in both LSD and HSD fed animals (Figure 5A).”

This is not true. On HSD it’s only 5% and not significant.

*Has been corrected.*

Why are Grn targets genes defined as ‘sugar up-regulated and grn-i downregulated” (e.g. Figure 6A) if it looks like Grn is rather activated on a LSD? Then the targets would be “sugar down-regulated”. Are the authors maybe looking at the wrong set of target genes? Is the Grn binding motif enriched in the set of genes that is up-regulated in LSD compared to HSD (ie the ones going down on the HSD)?

*Grain binding is only 25% on the genes downregulated by sugar feeding, compared to >50% of genes that are upregulated by sugar (Figure S1D vs. 1C). The inconsistency is hopefully now resolved by our tissue-specific data, showing both sugar-responsive and constitutive modes of Grain-mediated gene expression.*

Another option is that Grn activity is not regulated by sugar, and instead it constitutively drives mild expression of these target genes. In the HSD, other transcription factors such as sug become active and mask the effect of Grn. In that case, the target genes do not need to increase in the HSD condition compared to the LSD condition.

*Our new data is consistent with the conclusion that grain is a prominent regulator of lipogenic gene expression in the anterior part of the midgut, on both low and high sugar conditions (Figure 10). Our new qPCR data (Figure* 9*) is consistent with the proposal of the Reviewer that other sugar-regulated transcription factors override the effect of Grain, likely through activation of a more widespread lipogenic gene expression. We have now extended our Discussion in light of the new data obtained.*

3. The one transcriptional change that I can see in the manuscript which is large enough in magnitude that I can imagine it would lead to a change in protein levels is that of Aldh-III in the LSD, where it drops 2-fold upon Grn loss-of-function (Suppl. Fig. 4A). This might be biologically meaningful. Can Aldh-III levels be detected by western blots? Maybe all the other transcriptional effects are present but not biologically meaningful.

*We were not able to detect Aldh-III protein expression. However, we now show a localized downregulation in ACC LacZ reporter expression in the anterior part of the midgut.*

4. All the transcriptional changes shown in the manuscript are on whole-larval lysates, if I understand correctly. Maybe there are indeed large-magnitude changes going on in specific tissues that do not represent the bulk of the animal mass, and hence are getting diluted out when whole larvae are homogenized. Can some of these transcriptional changes be analyzed at a tissue-specific level, for instance with publicly available fluorescent transcriptional reporters (e.g. GFP transcript-traps in the genome, or synthetic constructs where genomic regions upstream of target genes were hooked up to GAL4?)

*Thank you for this excellent suggestion, which seems to have resolved some discrepancies in our original data. Indeed, the use of grain-GFP and ACC-LacZ show that Grain-mediated regulation of lipogenic gene expression occurs in a localized manner in the anterior part of the midgut (Figure 10).*

Minor Issues:

1. Fig 1B is unclear to me as currently shown. Are the binding motifs for the five drosophila GATA factors known? If so, it would be useful to show them, and hence how they differ so it becomes apparent why the grn motif matches best.

*We have added these motifs as Figure S1A, as requested.*

2. Fig 3C – the drop in expression of Hex-A upon grn LOF on HSD is very small in magnitude (5% drop?) and it is not even significant. It is a bit misleading as shown, with the x-axis starting at 120. This is true also for many other figures showing gene expression data. It would be more fair to show graphs with the y-axis starting at 0.

*Indeed, the drop in Hex-A expression is very small and not significant. We have now revised our conclusions regarding the glycolytic gene expression changes on HSD. We prefer to keep the box plot data as is, as the style is consistent with our earlier studies on related topics.*

3. Fig 3C most of the genes such as Gapdh1, Gapdh2 and PyK show no drop on the HSD at all upon Grn loss-of-function. It is misleading as stated that “many glycolytic genes… were expressed at a lower level in Grain-deficient animals, either on LSD or on both dietary conditions.” This is true for the LSD, but not for both dietary conditions.

*We have now moderated our conclusions regarding the glycolytic gene expression changes on HSD.*

4. Is the 30% drop in cbt upon Grn loss-of-function in the LSD shown in Fig 5A visible at the protein level?

*We have explored the protein expression of Cabut, as requested. Our preliminary data suggests that Cabut localization is posttranslationally regulated by sugar, which complicates the expression analysis (but is very interesting by itself). We prefer analysing Cabut protein regulation more thoroughly in a future study.*

5. The statement in the Results “Interestingly, cabut expression was significantly downregulated upon Grain depletion in both LSD and HSD fed animals (Figure 5A).” is not correct if in the HSD condition it’s dropping only 10% and not statistically significant.

*Thank you for pointing this out, it was indeed incorrect. Has been corrected now.*

6. All the changes in gene expression shown in Fig 6D are so minor in magnitude (not even a 2-fold change in any single gene), I doubt they are reflected at the protein level and hence of any biological meaning (unless there’s a large magnitude change in a specific tissue) ?

*We have now expanded our tissue specific analyses and show more prominent tissue-specific effects (see Figures 9 and 10).*

7. The citations of Fig. 6D and 5E are swapped in the Results section (page 8)

*Has been corrected.*

**Reviewer #4:**

In this study Kokki et al. report the role of grain, encoding a GATA transcription factor, in the regulation of the expression of several genes involved in sugar tolerance and metabolism. Trough in silico analysis authors showed that several genes that are upregulated by sugar are also grain target genes and many of these genes appear to be direct targets of grain as inferred by CHIP data.

Loss of function show that, genes normally downregulated in high sugar diets were upregulated upon grain knockdown and flies with reduced grain failed to pupariate an had impaired survival when fed a high sugar diet. Genes encoding for glycolytic enzymes are downregulated upon grain knockdown in high or low sugar diet conditions. Metabolic analysis of grain loss of function animals shows upregulation of TCA cycle intermediates, increased NAD+/NADH ratio and glucose and hexosamine pathway, most prominently in low sugar diet. Others sugar-responsive transcription factors, such as Cabut and Smox , are also regulated by grain.

In summary this paper uncovers a role for the GATA transcription factor Grain that in organismal glucose tolerance and systemic metabolism. However, most of the data represents the description of different phenotypes resulting from loss of function of grain and lacks mechanistic/functional work to begin to understand where and at which level Grain is working and how the metabolic phenotype are linked to the role of other sugar-responsive transcription factors and the how these phenotype relate to the organismal response of grain deficient animals to variations in dietary sugar levels.

Specific comments/questions:

1-What is the functional importance of grain when compared with sugarbabe?

*We have now added a direct comparison of Grain vs. Sugarbabe contribution on lipogenic gene expression (Figure 7). We have also added a comparison of their impact on TAG storage in the Discussion.*

2- Survival or fitness of larvae with grain deficiency appears selective to HSD. However, gene loss appears to impact metabolism in high and low sugar diet.

*Based on our tissue-specific data, Grain seems to have two distinct modes of action in different tissues (see Figures 9 and 10). Therefore a possible explanation is that the sugar-specific survival defects and the metabolic phenotypes (mainly on LSD) reflect these distinct activities. We have elaborated this in the revised Discussion.*

3- Figure 2a: what are the basal levels of grain normal dietary conditions. Throughout the study, low and high sugar diet conditions are presented but not normal diet conditions. This should be included

*The focus of our study is the understand the role of Grain with respect to changing dietary sugar. Therefore, we have chosen to use defined minimal experimental diets, where only dietary sugars are changing. It is not entirely clear to us, what is meant by “normal diet conditions”, since a number of different Drosophila diets are being used in research laboratories, each with distinct macronutrient composition. Understanding the role and regulation of Grain with respect to other nutrients is an interesting question, but beyond the scope of this study.*

4- How can the authors reconcile similar effects of grain loss in HSD and LSD. This is confusing to me.

*A reference to a specific Figure is lacking, so unfortunately interpreting this question is difficult. Based on our new tissue-specific data, Grain has both sugar-dependent and independent effects, which we have elaborated in the Discussion.*

5- Where is grain expressed?

*As requested, we have now analyzed tissue-specific expression of grain. It is most highly expressed in the midgut, especially the anterior part and displays lower, but sugar responsive, expression in the fat body and Malpighian tubules (Figures 9 and 10, Figure S7).*

6- From the results in figure 4 the authors conclude that High NAD+/NADH reflects impaired glycolysis and TCA cycle. However, most of the TCA cycle intermediates are elevated in grain mutants. How can the authors conclude that it is impaired? High NAD+/NADH could be a consequence of high mitochondrial respiration.

*Accumulation of TCA cycle intermediates can be caused by impaired flux through the cycle. However, this cannot be properly concluded using steady state metabolomics. As the reviewer correctly pointed out, high NAD+/NADH could be caused by a number of reasons, including high mitochondrial respiration. Therefore, we have now eliminated the speculation regarding impaired TCA cycle.*

7- Figure 7D: these results are not clear. Are all images taken at the same magnification? From the size of the nuclei, this appears not to be the case in some panels.

*Yes they are (scale bar included). There is some variability in nuclear and cell size between replicate samples, as the nuclei and cells grow through endoreplication during larval development. We have taken this into account in the experiment by normalizing the lipid droplet area by cell area.*

8- In all figures, please indicate for the statistical analysis, the value of the statistic and the degrees of freedom. Also, in the case of the positive interactions in the two-way ANOVA analysis the F of the interaction must be shown.

*In the case of ANOVA statistics, the Figure legends have been revised as requested. For details of RNA-seq and ChIP-seq analysis, detailed explanations are in the Materials & Methods.*

9- If “grn” is the gene must be in italics, please change that in all figures. The same for grn RNAi, in some figures is indicated like “grn RNAi”, but in others is only “grn”, the same nomenclature should be used in all cases.

*Has been corrected and unified, as requested.*

10- In all figures only one control is shown and is not clear if it’s the control for the driver or for the UAS, both controls should be shown at least in the supplementary figures. In the legends, control appears as tubG4> Trip control, also this is not clear. UAS-RNAi controls should be included.

*We have run UAS-RNAi controls on sugar tolerance (Figure 2B, Figure S2D) to further confirm the specificity of these data.*

11- Fig.2. Authors should indicate if they are measuring the levels of grain or the levels of grain target genes. Is the difference between mxl in LSD and HSD statistically significant?

*Figure 2A displays the expression of grain gene in mlx mutants. The difference between mlx^l^ in LSD and HSD is not statistically significant (has now been indicated).*

12- Fig. 3. C. Same as in figure 1, please defined what does CMP means. In the graph were the levels of Gapdh1 are shown, it seems to be a difference of *** between ctrl and grain RNAi in s HSD context, but it’s hard to see since the means are almost the same. I think there is a problem with the statistics there.

*Indeed the p value in Gapdh1 ctrl and grain RNAi in HSD was marked wrong, thank you for pointing it out. It has now been corrected. CPM stands for ‘counts per million’, we have now added the definition into the Figure legends.*

13-Fig. 4 Please checked the format of the genotypes, in same cases “control” is called “ctrl” and in this case is “cont”.

*Has been corrected as requested.*

14- Fig.5 The expression levels of cabut and smox appear to increase similarly in HSD in comparison to the LSD gene levels whether grain is there or not.

*This is indeed the case in the whole body mRNA samples. Our new tissue specific data (Fig 9) now shows that Grain contributes to sugar responsive expression of cabut and smox in the fat body.*

**Reviewer #5:**

Overall, I did like the paper. I found the study interesting, having found a novel role for the GATA TF Grain in nutrient sensing and involved in the Mondo/Mlx pathway (previously unknown). The fact that they found this previously unknown pathway through in silico motif analysis was also impressive. I do believe that the paper is up to standard for PLOS Genetics and only have a few revisions to suggest.

Here are my comments/revisions:

Major:

-I have concerns about how the data was analyzed and the significance of the data. In particular, Figures2C and 4B-G. A lot of the p-values calculated show significant differences, however the error bars on this data are particularly large and seem to overlap significantly. While the method suggests there were appropriate corrections made to the data, I find this troubling. Figure 4 in particular uses a very low N and I worry that a two-factor ANOVA may have a lot of false positives with the low N. With that, I would suggest an increased the N and would also like a power analysis of these data.

*The Reviewer pointed out the unrealistically low p-values in Figure 4. We have checked our calculations and have found a mistake in the original statistical analysis. After correction of the analysis, most metabolites that earlier showed statistically significant changes, remain significant, although with higher p-values. We apologize for making the mistake and thank the Reviewer for raising the concern.*

*The number of biological replicates in the metabolomics experiment is five (not four as originally labeled) for three samples and four for one sample, with 20 animals included into each replicate. Therefore, we believe the risk of false positives due to sampling bias is low. Although TCA cycle metabolites display modest fold changes, with borderline p-values (p<0.08), the fact that several metabolites in the same pathway display similar changes increase the reliability of the data. Therefore, we have chosen to keep be TCA cycle data in the manuscript, but remained cautious in the interpretation. If the Reviewer or Editor prefers the TCA cycle data to be eliminated, we are happy to do it.*

-I found figure 3 to be very confusing with the flow of the rest of the paper. Figure 3A and B focus on the genes that were upregulate in control HSD flies but also downregulated in Grain-RNAi. However, the rest of the paper focuses on Grain's role in low sugar diet conditions. With that, I am more interested in what genes were enriched in control HSD but downregulated in grain LSD and compared with both figures 3 A and B.

*Data in Figure 3A displays genes that are upregulated by HSD feeding (the original gene set including high percentage of Grain direct targets) and display significant downregulation by Grain knockdown on either (or both) dietary conditions. So, this dataset includes the genes that are downregulated by grain knockdown on LSD. As based on our new data (Figure 9) Grain appears to have tissue-specific roles on regulating some target genes on both LSD and HSD conditions, we think including Grain-dependent genes on both dietary conditions into the analysis of Figure 3A is justifiable. We have now clarified the choice of the gene set in the amended manuscript.*

-Figure 3B seems unnecessary to the main paper and should be moved to the supplement. The authors suggest in 3B that gene expression of grain targets compared to all genes in their RNAseq analysis have a tissue specific enrichment in the midgut. However, the analysis ends there. Figure 3B also appears biased to me, since it is comparing only the genes upregulated by control HSD but downregulated by grain HSD. I think a more appropriate analysis would be a comparison of tissue enrichment in all genes that were down regulated by grain-RNAi to all genes in the set. If this is an important finding to the paper, I would also like to see if the phenotypes seen in Figure 2 could be reproduced using a midgut specific grain knockdown or knockout. This would suggest that the metabolic phenotypes at play in the rest of the paper are due to the tissue specific effects of GATA in the midgut.

*As requested, Figure 3B has been moved to the supplement (Figure S7). We have now significantly strengthened the tissue-specific analysis of grain expression and function. Our data shows that grain displays highest expression in the midgut, but this is not sugar responsive. In contrast, grain expression in fat body and Malpighian tubules is activated by sugar feeding. Our target gene analysis further imply that Grain contributes to metabolic gene regulation in sugar dependent and independent manner in different tissues. This potentially explains, why certain phenotypes are seen on HSD, while others on LSD (see Figures 9 and 10). We have now elaborated our Discussion in light of this new data.*

-In figure 5D, I would like to see the expression profiles of the sugarbabe mutated larvae next to the grain-rnai data, as this would help visualize the similarity in gene regulation between grain and sugarbabe that the authors note.

*This data has been added, as requested (Figure 6).*

Minor:

-For Figure 4A, I think it would be helpful to color code the change in expression of the metabolites that were impacted by grain knockdown (i.e. blue shaded metabolites were lower in grain-RNAi HSD compared to control HSD and red shaded metabolites were higher in grain-RNAi HSD compared to control HSD).

*We have changed the color coding as suggested.*
