# Supplementary material for: Metabolic gene regulation by Drosophila GATA transcription factor Grain
Source: PLoS Genet. 2021 Oct 11;17(10):e1009855. doi: 10.1371/journal.pgen.1009855 (PMC8530363; doi:10.1371/journal.pgen.1009855)
Supplement: Attachment — Submitted filename: Kokki_point by point.docx [file pgen.1009855.s013.docx]

**Point-by-point response Kokki, Lamichane et al.**

Some edits to the text will improve the manuscript:

1. The low sugar diet is essentially a no sugar diet while the high sugar diet is 10% sucrose (lines 319-322 in the methods). This information should also be provided in the main text and/or the first figure legend to give context to the results (most readers will read the methods section last (if at all)).

*As suggested, we have added this information info to the first paragraph of the Results section. This information is already present in all figure legends.*

2. It is possible that a diet so low in sugar leads not only to sugar deficiency but also to protein to sugar mismatch as a metabolic challenge (see for example Skorupa et al., 2008, Aging Cell). This should be considered briefly in the Discussion section.

*We have now added the reference and mentioned the importance of protein/sugar balance in the Discussion.*

3. Much of the data in figures 1 through 8 is from whole-animal measurements of metabolites or gene expression in control larvae or larvae with ubiquitious knockdown of grain. It would be helpful to the reader to point out in each section of the results that the data being describe are from measurements from whole larvae.

*This information was already present in most places of the results, but we have now added it also to the remaining sections.*

4. The authors were unable to carry out epistasis experiments with grain and Smox / cabut, so we do not yet know whether altered expression of these transcription factors upon grain knockdown underlies some of the metabolic / gene expression phenotypes described in this article. Nonetheless, this should be mentioned as a possibility in the Discussion.

*We have added a sentence in the Discussion indicating the need for future genetic epistasis experiments to test the contribution of Grain targets on sugar tolerance.*

Comments on figures:
1. Graphs in Figure 4B-4G lack y-axis labels.
*Have been added, as requested.*

2. For Figure 8A, if they are known, units should be added to the y-axis label. (µg TAG per mg protein, for example)
*Units have been added, as requested.*

3. Please define "CLI" for Figure 8C.

*As suggested, we have now defined CLI in the legend of Figure 8.*

Minor text edits:
1. line 236 - extra ")" symbol

*Has been deleted.*

2. line 281 - "a downstream of Dawdle/Smox" - is the word "target" omitted?

*We have now added the word “target”.*
